# Integrative Multi-Omics Analysis and Experimental Validation Identify SPOP as a Prognostic Biomarker and Immune Regulator in Lung Adenocarcinoma

**DOI:** 10.7150/jca.111751

**Published:** 2025-06-23

**Authors:** Yu Wang, Tao Jiang, Ziyou Lin, Peijun Dai, Wenxin Wei, Chengyuan Dong, Xuelin Zhang, Zhifeng Zhang

**Affiliations:** 1 Department of Hepatic Surgery, Third Affiliated Hospital of Naval Medical University, Shanghai, China.; 2 Department of orthopedics, Third Affiliated Hospital of Naval Medical University, Shanghai, China.; 3 Department of thoracic surgery, Huadong hospital, Shanghai, China.; 4 Shanghai TCM-Integrated Hospital, Shanghai university of TCM, Shanghai, China.; 5 Tongji University School of Medicine, Shanghai, China.

**Keywords:** SPOP, lung adenocarcinoma, tumor immunity, prognostic biomarker

## Abstract

**Background:** The speckle-type POZ protein (SPOP) has emerged as an important regulator of protein degradation in various cancers. However, the precise role of SPOP in lung adenocarcinoma (LUAD) remains unclear, particularly in relation to its expression patterns, prognostic significance, and potential as a therapeutic target. This study aimed to investigate the expression, prognostic value, and biological functions of SPOP in LUAD, and to explore its potential as a biomarker for personalized treatment strategies.

**Methods:** We performed a comprehensive analysis of SPOP expression using multiple public datasets, including TCGA, TCGA-GTEx, and GEO. Survival analyses were conducted through Cox regression and Kaplan-Meier methods to assess the prognostic significance of SPOP in LUAD. Gene Set Variation Analysis (GSVA) and Gene Set Enrichment Analysis (GSEA) were employed to uncover biological pathways associated with SPOP expression. Immune microenvironment analysis and drug sensitivity data from the GDSC database were used to explore the potential role of SPOP in immune modulation and therapeutic response. The biological role of SPOP in LUAD was further explored through molecular docking analysis and experimental validation.

**Results:** SPOP expression was significantly reduced in LUAD compared to normal tissues, with lower expression correlating with poor overall survival (OS), disease-specific survival (DSS), and progression-free interval (PFI). Cox regression analysis confirmed that SPOP is an independent prognostic factor for LUAD. Functional analyses revealed that low SPOP expression was associated with disrupted immune regulation and altered metabolic pathways, potentially driving tumor progression. Immune profiling identified significant correlations between SPOP expression and immune cell recruitment, inflammatory signaling, and LUAD subtypes. Drug sensitivity analysis suggested that low SPOP expression is linked to increased sensitivity to zibotentan and 5-fluorouracil. Additionally, molecular docking analysis revealed key interaction sites between SPOP and NANOG, and SPOP knockdown in A549 and T24 cells resulted in downregulation of immune markers CD47 and CD155.

**Conclusion:** SPOP is a reliable independent prognostic biomarker in LUAD, influencing tumor progression, immune microenvironment, and therapeutic response. Our findings support the potential of SPOP as a novel therapeutic target for personalized treatment strategies in LUAD.

## 1. Introduction

Lung cancer remains the second most common malignancy worldwide and a leading cause of cancer-related deaths 1. Despite significant advances in diagnostic and therapeutic strategies in recent years, the five-year survival rate for LUAD patients remains under 20% 2, 3. The emergence of immunotherapy has significantly changed the treatment landscape, especially for metastatic diseases, but the variable response rates highlight the complexity of tumor-immune interactions within the tumor microenvironment (TME) 4-6. This heterogeneity in treatment responses underscores the urgent need for identifying new molecular biomarkers to predict therapeutic responses and discover novel therapeutic targets.

SPOP is a Cullin 3-based E3 ubiquitin ligase adaptor protein, and its multifunctionality in tumor biology has made it a prominent focus of cancer research 7-9. SPOP comprises three primary structural domains: the N-terminal MATH domain, which facilitates substrate recognition, the BTB domain that supports cullin3 binding and dimerization, and the C-terminal nuclear localization signal 7. These domains enable SPOP to perform diverse roles across different cancer types. Recent studies have shown that mutations or downregulation of SPOP are closely linked to tumorigenesis in various cancers. SPOP substrates include key oncogenic factors such as AR, PD-L1, c-Myc, BRD4, ERα, SRC3, Gli2/3, DEK, TRIM24, cyclin E1, CDC20, SENP7, CDCA5, STAT3, and EglN2 10-24. SPOP impacts inflammation by regulating the TLR-MyD88-NF-κB signaling axis 25. These findings suggest that SPOP may serve as a critical link between tumor development and immune response. However, the molecular heterogeneity of SPOP function across different cancers highlights the need for multi-omics analyses to establish consistent standards.

In this study, we explored the role of SPOP in cancer, focusing on its expression patterns, prognostic significance, and its relationship with immune cell infiltration in LUAD. Additionally, we investigated the correlation between SPOP expression and various immune-related parameters. Through this systematic approach, we aim to elucidate the potential role of SPOP in tumor immune regulation and evaluate its impact on the immune therapy response in LUAD patients.

## 2. Materials and Methods

### 2.1. Data sources

This study utilized gene expression data from the corrected TCGA database, sourced from the EBPlusPlusAdjustPANCAN_IlluminaHiSeq_RNASeqV2.geneExp.tsv file provided by PanCanAtlas. For patient inclusion, the following criteria were applied: only patients with a confirmed diagnosis of tumor, specifically those with digestive system-related cancers, were included. All patients were required to be adults (≥18 years old), and those with other severe comorbidities (such as cardiovascular diseases, immune system disorders, or other types of cancer) were excluded. Additionally, only tumor samples with complete clinical follow-up data were included to ensure the integrity and accuracy of the data. To eliminate dimensional differences between samples, the data were standardized by calculating the Z-score using the formula (x-μ)/σ(x - \mu) / \sigma(x-μ)/σ. Outliers, defined as Z-scores less than -3 or greater than 3, were removed. After data cleaning, only tumors with at least three normal samples were included in the analysis.

### 2.2. Differential expression analysis

Differential expression of SPOP between tumor and normal tissues was analyzed using the "limma" R package. GSE72094 and GSE41271 were employed to validate the results.

### 2.3. Prognostic analysis

The prognostic significance of SPOP in LUAD patients was assessed using both univariate and multivariate Cox proportional hazards regression models. Kaplan-Meier survival analysis was performed to evaluate OS, DSS, and PFI between high and low SPOP expression groups.

### 2.4. Functional annotation and pathway analysis

GSVA was performed using the "clusterProfiler" R package to identify biological processes and signaling pathways associated with SPOP. GSEA was conducted to comprehensively assess SPOP-related pathway signatures.

### 2.5. Immune profile analysis

The role of SPOP in various stages of the antitumor immune response was further explored using the Tracking TIP tool.

### 2.6. Drug sensitivity analysis

The relationship between SPOP expression and drug sensitivity was investigated using data from the GDSC database and cMAP. Statistical analyses were performed with the "pRRophetic" R package, combining p-values and effect sizes. Stratified analyses were carried out for specific therapeutic agents to validate drug response patterns associated with SPOP expression levels.

### 2.7. Cell lines and culture

A549 and T24 cell lines were purchased from the American Type Culture Collection (ATCC). Cells were cultured in RPMI-1640 medium (Gibco, USA) supplemented with 10% fetal bovine serum (FBS, Gibco, USA) and 1% penicillin-streptomycin (Gibco, USA) at 37°C in a 5% CO2 incubator.

### 2.8. SPOP knockdown

To screen and validate the regulation of specific target genes, two shRNA sequences were designed for gene silencing, with the sequences as follows: sh1: CTCCTACATGTGGACCATCAA and sh2: CACAAGGCTATCTTAGCAGCT. These shRNA sequences were synthesized by Shanghai Shenggong and were used in subsequent gene knockdown experiments.

### 2.9. RNA extraction and quantitative PCR (qPCR)

Total RNA was extracted from cells using the RNAiso Plus reagent (Takara), and reverse transcription was performed using the PrimeScript RT reagent kit (Takara). Quantitative PCR was performed using SYBR Premix Ex Taq II (Takara). Gene expression was normalized to GAPDH. The primers used for PCR are as follows:

SPOP: Forward (5'-GCTGCTGAGGAAGAGGACAT-3'),

Reverse (5'-AGGAGTGAGGAAGTGGGAGT-3');

GAPDH: Forward (5'-GGAGCGAGATCCCTCCAAAAT-3'),

Reverse (5'-GGCTGTTGTCATACTTCTCATGG-3').

### 2.10. Flow cytometry analysis

For cell surface marker analysis, cells were harvested, washed with PBS, and stained with specific antibodies against CD47 (PE-conjugated, BioLegend) and CD155 (APC-conjugated, BioLegend) for 30 minutes at 4°C. After staining, cells were washed and analyzed using a flow cytometer (BD Biosciences). Data were analyzed using FlowJo software.

### 2.11. Visualizing SPOP-NANOG interaction in PyMOL

In PyMOL, import the PDB files of SPOP and NANOG, and visualize the interaction between them by displaying the amino acid residues involved in their binding. This will help further illustrate how the proteins interact with each other.

### 2.12. Statistical analysis

All statistical analyses were performed using R software (version 4.2.0). Continuous variables were compared using Student's t-test, while categorical variables were compared using the chi-square test. A p-value of <0.05 was considered statistically significant for all analyses.

## 3. Results

### 3.1. Analysis of SPOP expression across cancer types

**Figure 1:**
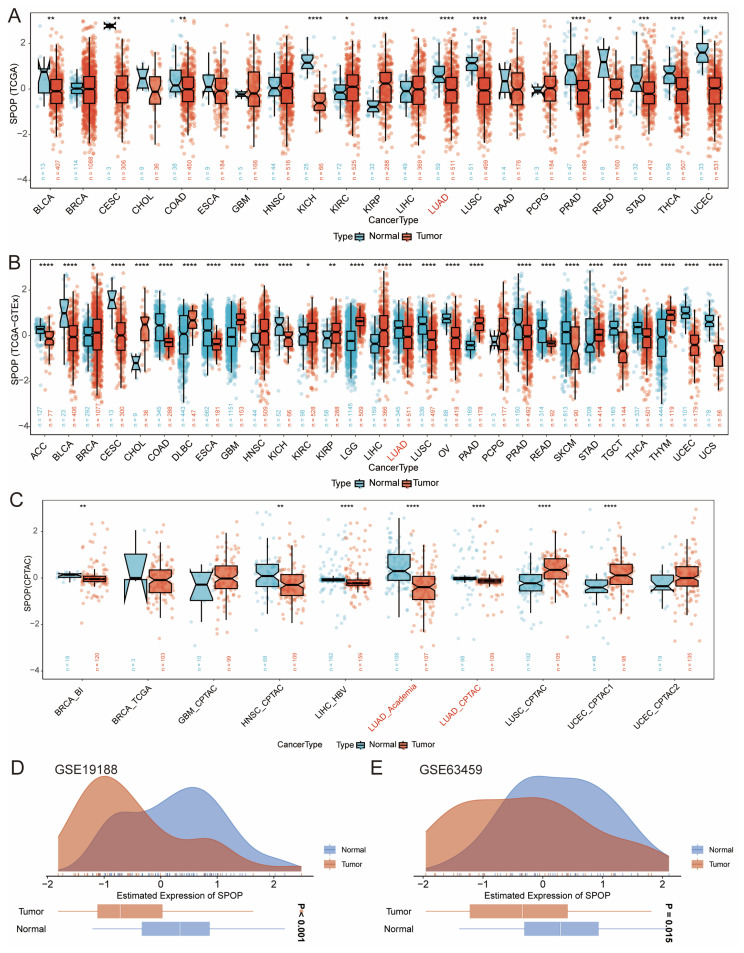
Pan-cancer analysis of SPOP expression patterns. (A) Comparison of SPOP expression between tumor and adjacent normal tissues across cancer types (TCGA). (B) Comprehensive analysis of SPOP expression using combined TCGA and GTEx data. (C) SPOP expression patterns in protein databases. (D-E) Validation of SPOP expression distribution in independent cohorts using GSE19188 and GSE63459 datasets. For all panels, significance levels are indicated as: *P<0.05, **P<0.01, ***P<0.001, ****P<0.0001.

We conducted an in-depth analysis of SPOP expression using TCGA data (**Figure 1A**), integrated TCGA-GTEx analysis (**Figure 1B**), and protein datasets (**Figure 1C**). The results demonstrated consistently lower levels of SPOP expression at both mRNA and protein levels in LUAD and lung squamous cell carcinoma (LUSC) compared to normal tissues, with particularly significant changes observed in LUAD. To further validate these findings, we performed external validation using the GSE19188 and GSE63459 datasets (**Figure 1D-E**), confirming the robust quality and consistency of SPOP expression patterns in LUAD.

### 3.2. Prognostic significance of SPOP in cancer survival analysis

**Figure 2:**
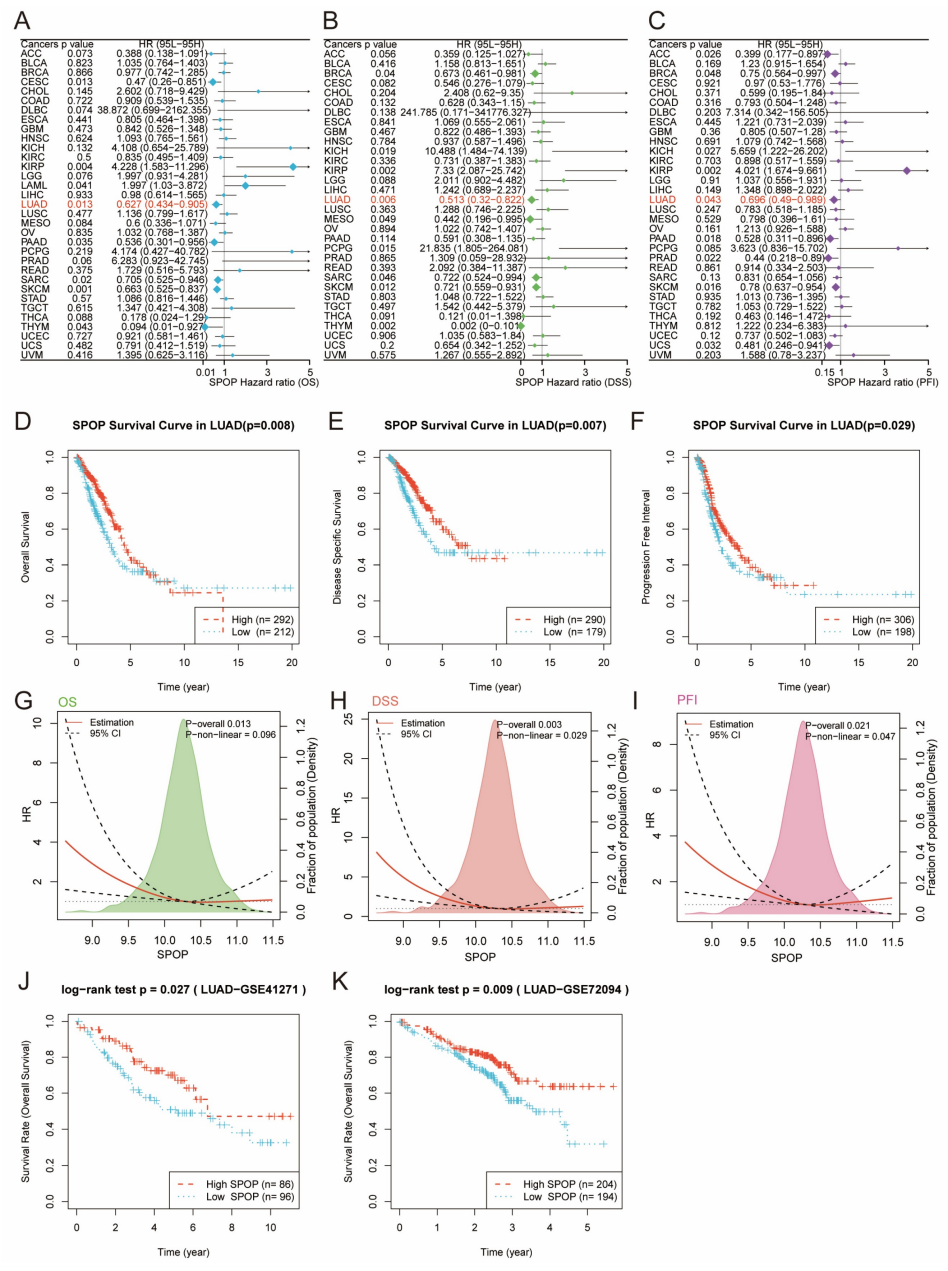
SPOP prognostic value across cancer types and detailed analysis in LUAD. Forest plots showing the prognostic significance of SPOP across different cancer types through univariate Cox regression analysis for OS (A), DSS (B), and PFI (C). Kaplan-Meier survival curves comparing high and low SPOP expression groups in LUAD patients for OS (D), DSS (E), and PFI (F). Non-linear correlation analyses between SPOP expression and survival risk, showing both overall and non-linear associations for OS (G), DSS (H), and PFI (I). Validation of SPOP prognostic value in external LUAD cohorts from GSE41271 (J) and GSE72094 (K) datasets.

We further evaluated the prognostic value of SPOP across different cancer types. Univariate Cox regression analysis revealed that low SPOP expression was significantly associated with poor survival outcomes, including OS, DSS, and PFI in LUAD patients (**Figure 2A-C**). To further validate these findings, Kaplan-Meier survival analysis was conducted on LUAD patients. The results demonstrated that patients with low SPOP expression had significantly shorter OS, DSS, and PFI compared to those with high expression (**Figure 2D-F**). Additionally, further exploration of the relationship between SPOP expression and survival risk revealed significant linear associations for OS, DSS, and PFI, suggesting that SPOP can serve as a stable marker for assessing patient prognosis (**Figure 2G-I**). To validate these results, we analyzed two independent LUAD cohorts from the GEO database (GSE41271 and GSE72094). Consistent with the initial findings, both validation cohorts confirmed that high SPOP expression was associated with better overall survival (**Figure 2J-K**). Collectively, these results highlight SPOP as a reliable prognostic biomarker in LUAD.

### 3.3. Independent prognostic value of SPOP in LUAD

**Figure 3:**
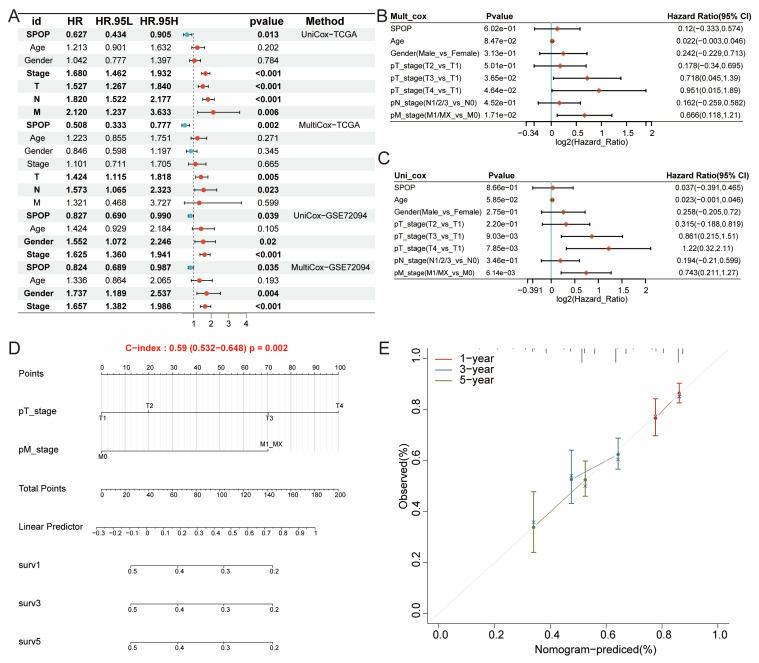
Independent prognostic factor in LUAD. (A-C) Independent prognostic features of SPOP expression in TCGA and GSE72094 cohorts. (D) Nomogram predicting survival probability based on SPOP expression and pathological stages. (E) Calibration curves showing the predicted 1-, 3-, and 5-year survival rates.

Building on the differential expression and significant prognostic value of SPOP in LUAD, we proposed that SPOP could serve as an independent clinical prognostic factor. Both univariate and multivariate Cox regression analyses validated this hypothesis, identifying SPOP, along with stage, T, M, and N classifications, as independent prognostic factors. This significance was further confirmed in the independent GSE72094 cohort (**Figure 3A-C**). To enhance the clinical utility of these findings, a nomogram was developed, showing strong predictive performance (**Figure 3D**). Calibration curves for 1-, 3-, and 5-year survival predictions revealed excellent concordance between the nomogram prediction and observed outcomes, further confirming the model reliability (**Figure 3E**). Together, these findings establish SPOP as a robust, independent prognostic biomarker in LUAD, with promising potential for clinical risk stratification and treatment planning.

### 3.4. Biological role of low SPOP expression in LUAD

**Figure 4:**
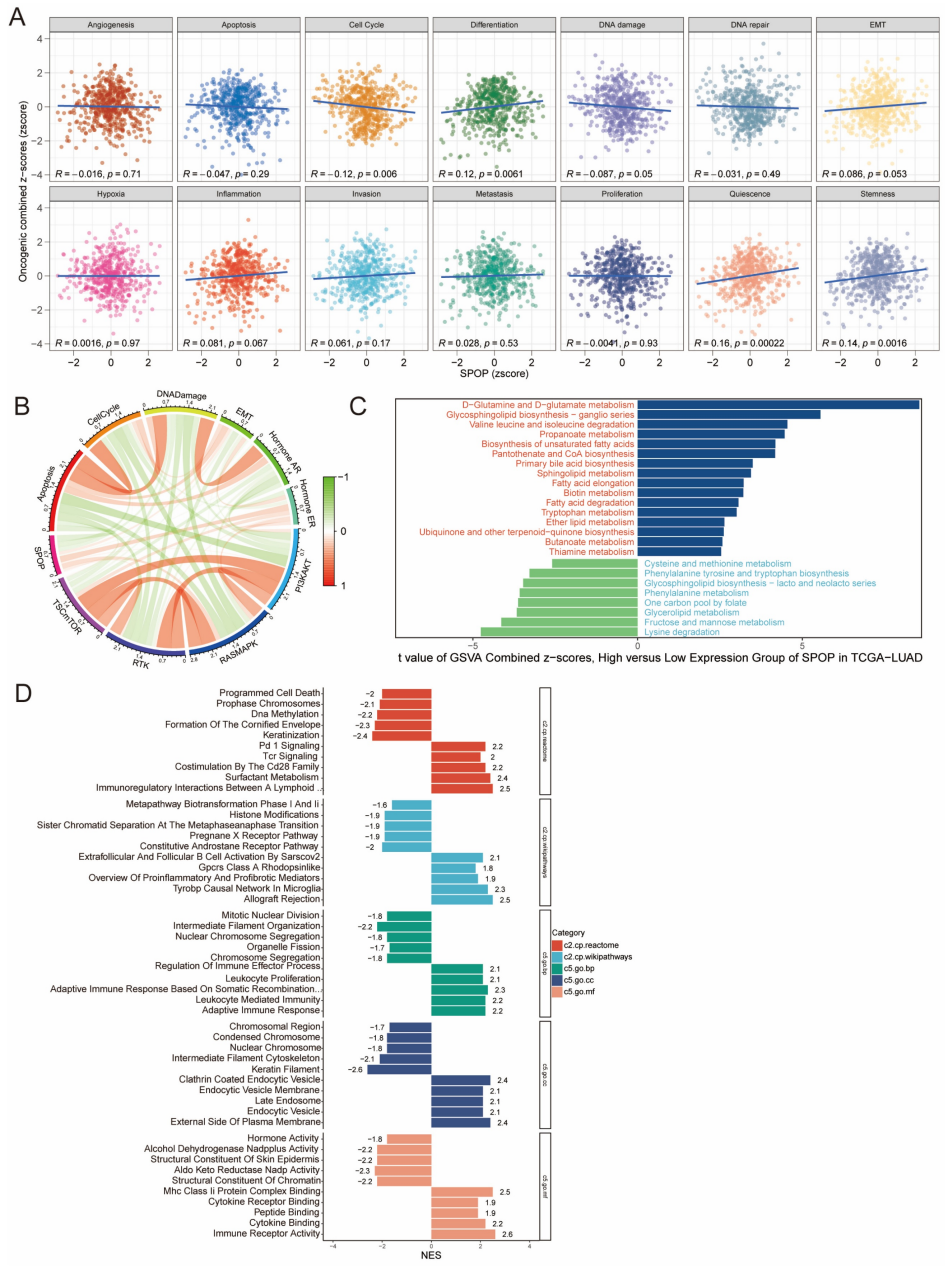
Functional analysis of SPOP in LUAD. (A) Correlation between SPOP expression and 14 hallmark cancer pathways. (B) Circos plot showing interactions between SPOP and cancer pathways. (C) GSVA analysis comparing pathway enrichment between high and low SPOP expression groups. (D) GSEA analysis of enriched pathways across functional categories with normalized enrichment scores.

To gain deeper insights into the biological functions of SPOP in LUAD, we analyzed the correlation between SPOP expression and GSVA scores across 14 hallmark cancer-related pathways. The results revealed a significant negative correlation between SPOP expression and both the cell cycle and DNA damage, while showing significant positive correlations with differentiation, quiescence, and stemness (**Figure 4A-B**). To further investigate the underlying biological mechanisms, we performed GSVA analysis. The high-expression group exhibited significant enrichment in pathways associated with D-glutamine and D-glutamate metabolism, glycosphingolipid biosynthesis, and various amino acid metabolic processes. In contrast, the low SPOP expression group showed notable enrichment in pathways involved in cysteine and methionine metabolism, glycerolipid metabolism, and lysine degradation (**Figure 4C**). GSEA analysis further revealed significant enrichment in processes related to the endocytic vesicle membrane and plasma membrane, as well as in adaptive immune response and leukocyte-mediated immunity (**Figure 4D**). Low SPOP expression in LUAD is associated with impaired immune regulation, altered cellular processes, and metabolic pathways, potentially contributing to tumor progression.

### 3.5. Immune landscape of SPOP expression in LUAD

**Figure 5:**
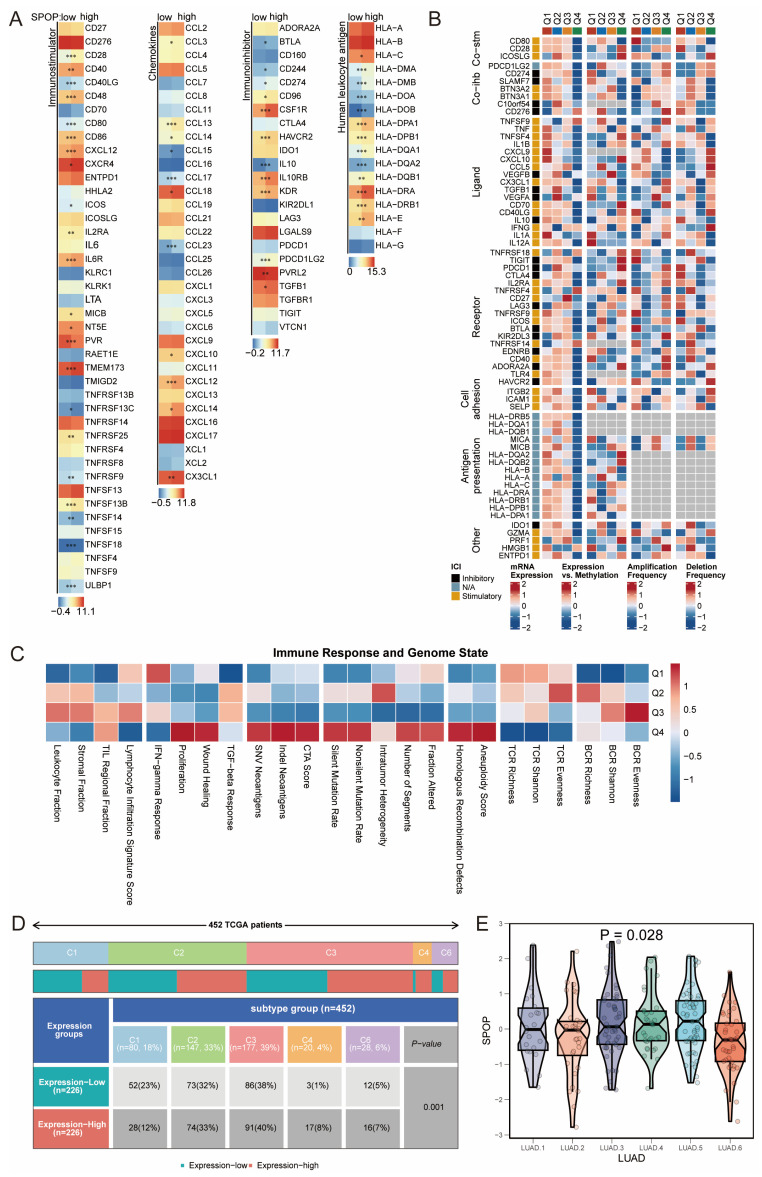
Immune Microenvironment Analysis. (A) Heatmap showing the correlation of immune checkpoints between SPOP-high and SPOP-low groups. (B) Association of SPOP expression with molecular characteristics of immune-related genes in LUAD (mRNA expression, methylation, amplification, and deletion). (C) Heatmap depicting the relationship between SPOP expression quartiles (Q1-Q4) and immune response/genomic states. (D) Distribution of SPOP expression levels across molecular subtypes in LUAD. (E) Violin plot showing significant heterogeneity of SPOP expression across six LUAD subtypes.

To further elucidate the role of SPOP in the immune microenvironment, we conducted an analysis of immune-related genes. The results showed significant upregulation of genes such as CD28, CD40, CD48, CD80, CD86, CXCR4, CXCL12, and TMEM173 in the SPOP-high expression group. Several HLA family members, particularly HLA-DMA, HLA-DRA, and HLA-DPA1, were also significantly upregulated. In contrast, SPOP expression was negatively correlated with genes like CCL15, TNFRSF13C, TNFRSF9, and TNFRSF14 (**Figure 5A**). Methylation status and copy number variations significantly influenced the regulatory effects of SPOP on the expression of HLA family members (**Figure 5B**). Assessment of immune response and genomic states revealed significant correlations between immune infiltration, TCR richness, and BCR characteristics (**Figure 5C**). Analysis of molecular subtypes showed significant differences in SPOP expression across the six LUAD subtypes (C1-C6), with a higher proportion of the C1 subtype (tissue repair type) in the SPOP-low expression group (**Figure 5D-E**). This subtype was associated with increased expression of angiogenesis genes, higher proliferation rates, and a Th2-biased acquired immune infiltration pattern.

### 3.6. SPOP in cancer immunity and drug response of LUAD

**Figure 6:**
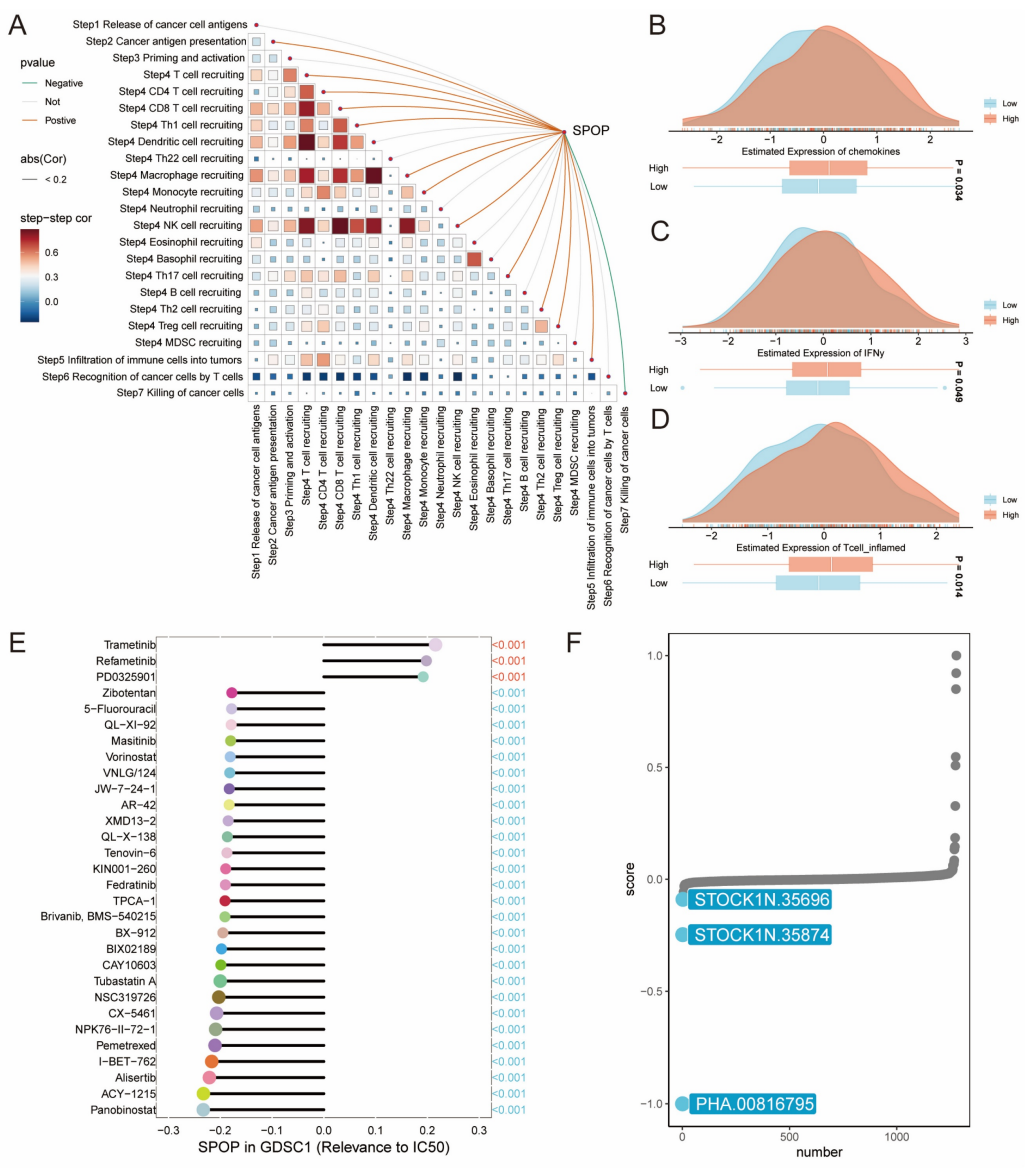
Cancer-immunity cycle and drug response. (A) Correlation matrix showing the relationship between SPOP expression and different steps of cancer-immunity cycle. (B-D) Density plots comparing expression levels of chemokines, IFNγ, and T cell inflammation signatures between SPOP-high and SPOP-low groups. (E) Drug sensitivity analysis based on GDSC database showing correlations between SPOP expression and drug response. (F) Identification of potential therapeutic compounds from connectivity map (cMAP) analysis that could modulate SPOP-associated pathways.

To comprehensively understand the role of SPOP in antitumor immunity and drug response, we conducted a stepwise analysis of the cancer-immunity cycle and drug sensitivity. The correlation matrix showed that SPOP expression was significantly positively associated with the recruitment of CD8+ T cells, CD4+ T cells, and Th1 cells, while negatively correlated with T cell recognition of cancer cells (**Figure 6A**). Further analysis of immune-related characteristics revealed that the SPOP-high group exhibited significantly elevated expression of chemokines, IFNγ, and T cell inflammation signatures compared to the SPOP-low group (**Figure 6B-D**). Drug sensitivity analysis using the GDSC database demonstrated a significant correlation between SPOP expression and drug response. Notably, trametinib, refametinib, and PD0325901 showed positive correlations with SPOP expression, suggesting potential resistance in SPOP-high tumors. In contrast, compounds such as zibotentan, 5-fluorouracil (5-FU), and QL-XI-92 were negatively correlated with SPOP expression, indicating enhanced sensitivity (**Figure 6E**). Additionally, connectivity map (cMAP) analysis identified three potential compounds (PHA.00816795, STOCK1N.35696, and STOCK1N.35874) that may target SPOP-associated pathways (**Figure 6F**). SPOP expression levels could serve as a predictive marker for drug response in LUAD and provide potential therapeutic strategies for distinct SPOP expression subgroups.

### 3.7. Experimental validation

**Figure 7:**
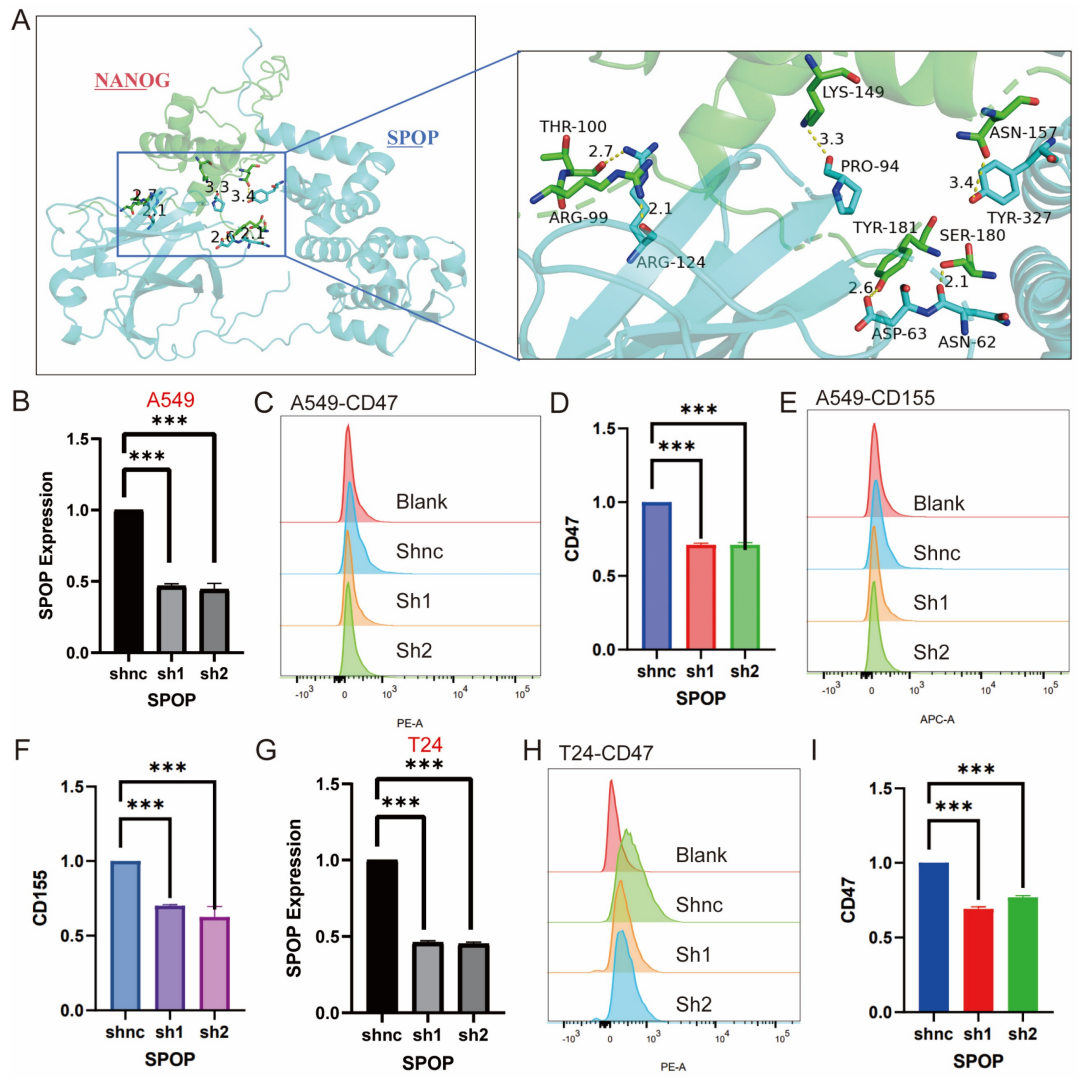
Effects of SPOP Knockdown on mRNA Expression and Cell Surface Marker Profiles in A549 and T24 Cells. (A) Structural interaction model between SPOP and NANOG, with an enlarged region showing key amino acid residues in NANOG (e.g., Arg99, Thr100, Lys149) interacting with SPOP. (B) mRNA expression analysis of SPOP knockdown in A549 cells. (C-D) Flow cytometry analysis of CD47 expression in A549 cells after SPOP knockdown. (E-F) Flow cytometry analysis of CD155 expression in A549 cells after SPOP knockdown. (G) mRNA expression analysis of SPOP knockdown in T24 cells. (H-I) Flow cytometry analysis of CD47 expression in T24 cells after SPOP knockdown.

Based on the above results and literature reporting the role of SPOP in regulating tumor stemness, we further investigated the key interaction sites between SPOP and NANOG 26. Through molecular docking analysis, we identified specific amino acid residues involved in the interaction between the SPOP and NANOG proteins. Specifically, the 124th ARG residue of SPOP forms two hydrogen bonds with the 99th ARG and 100th THR residues of NANOG, with bond lengths of 2.1 Å and 2.7 Å, respectively. Additionally, the 94th PRO residue of SPOP interacts with the 124th LYS residue of NANOG via a hydrogen bond with a length of 3.3 Å. The 62nd ASN and 63rd ASP residues of SPOP form hydrogen bonds with the 180th SER and 181st TYR residues of NANOG, with bond lengths of 2.1 Å and 2.6 Å, respectively (**Figure 7A**). Mutations at these sites may potentially affect the degradation of NANOG by SPOP, making these findings a critical area for future stem cell research.

Furthermore, while it has been reported that SPOP degrades the immune checkpoint PDL1 27, its regulation of other immune checkpoints remains unknown. Based on bioinformatics analysis, we experimentally explored the effects of SPOP knockdown on immune markers in A549 and T24 cells. In A549 cells, knockdown of SPOP significantly decreased the expression levels of CD47 and CD155, while no significant change in CD276 expression was observed (**Figure 7B-F**). In T24 cells, SPOP knockdown significantly reduced CD47 expression, but no changes were observed for CD155 and CD276 (**Figure 7G-I**). These findings provide important insights for further investigating the role of SPOP in tumor immune evasion and support its potential as a therapeutic target.

## 4. Discussion

Cancer remains one of the most pressing challenges in global health, with its incidence and mortality rates continuing to rise 28. Among various malignancies, LUAD is the leading cause of cancer-related deaths worldwide. Despite advancements in diagnostic imaging, molecular profiling, and therapeutic interventions, including targeted therapies and immunotherapies, the overall prognosis for LUAD patients remains poor 29, 30. Key contributing factors include delayed diagnosis, high recurrence rates, and a propensity for distant metastases 31.

The TME, defined by intricate interactions between cancer and immune cells, plays a critical role in the progression, metastasis, and immune evasion of LUAD 32. Although immunotherapy has transformed the treatment landscape for LUAD, its clinical success is often limited by inconsistent response rates and the development of resistance 33-35. These issues highlight the complexity of tumor-immune dynamics and underscore the urgent need to identify novel biomarkers and therapeutic targets to improve the efficacy of immunotherapy.

SPOP has emerged as a critical regulator of protein degradation and cellular homeostasis. The role of SPOP in cancer biology has been extensively studied, particularly in prostate cancer, where SPOP mutations are found in 6-15% of cases 36. These mutations primarily affect the MATH domain, impairing substrate recognition and disrupting the degradation of key oncogenic proteins such as the androgen receptor and BRD4, thereby driving tumorigenesis 37. In endometrial cancer, SPOP mutations alter estrogen receptor-α stability and enhance BET protein degradation, SPOP also regulates multiple substrates, including SRC-3, progesterone receptor, and c-Myc, to exert tumor-suppressive effects 12, 38-40. Interestingly, in clear-cell renal cell carcinoma, SPOP displays oncogenic properties by promoting the degradation of tumor suppressors such as PTEN and DUSP7, underscoring its context-dependent functions in cancer 41, 42.

This study provides a comprehensive analysis of SPOP in LUAD, establishing its role as a significant prognostic biomarker and immune modulator. Multi-cohort validation using TCGA, GTEx, and GEO datasets demonstrated that high SPOP expression correlates with improved OS, DFS, PFS. These associations were consistently validated across independent cohorts, and multivariate analyses confirmed that SPOP remains an independent prognostic factor even after adjusting for conventional clinical parameters. These findings highlight the robust prognostic value of SPOP in LUAD and its potential utility in guiding clinical decision-making.

Mechanistic insights revealed that SPOP plays a multifaceted role in LUAD by modulating key cellular processes and the immune microenvironment. GSVA and GSEA analysis identified significant enrichment of pathways related to immune response, metabolic regulation, and cellular membrane functions in tumors with high SPOP expression. Conversely, pathways associated with cell cycle progression and DNA damage repair were negatively correlated with SPOP expression, suggesting that SPOP exerts tumor-suppressive effects through mechanisms such as metabolic reprogramming and cell cycle inhibition. High SPOP expression was also associated with cellular differentiation and quiescence, further supporting its role in tumor suppression. High SPOP expression was associated with upregulation of immune-related genes, including CD28, CD40, and various chemokines, which are critical for immune cell activation and recruitment. Analysis of the cancer-immunity cycle revealed a positive correlation between SPOP and T cell recruitment and activation, particularly in CD8+ and CD4+ T cells, which are essential for anti-tumor immunity. Recent studies have provided insights into the molecular mechanisms underlying the immune-regulatory functions of SPOP 23. Additionally, SPOP mutations have been implicated in tumor immune escape by regulating the IRF1-PD-L1 axis, further highlighting its critical role in immune modulation 43. The therapeutic implications of these findings are significant.

Drug sensitivity analyses revealed that SPOP expression levels influence the response to various therapeutic agents. Recent advancements in targeting the ubiquitin-proteasome system have opened new avenues for cancer therapy 44. The development of SPOP inhibitors, and the discovery of SPOP-mediated non-degradative ubiquitination of BRAF provide promising strategies for therapeutic intervention 45. Notably, Maprotiline, identified as a PD-L1 regulator through SPOP targeting, has demonstrated synergistic efficacy with anti-CTLA4 therapy in preclinical models of colorectal and lung cancer, underscoring its translational potential 46.

This study has several limitations. First, the precise molecular pathways through which SPOP regulates immune responses in LUAD remain to be fully elucidated. Future studies should focus on experimental investigations to uncover the underlying mechanisms. Second, the tissue-specific functions and mutation-driven effects of SPOP warrant in-depth exploration to develop tailored therapeutic strategies. By elucidating its multifaceted roles, this research provides a foundation for future studies aimed at leveraging SPOP as a biomarker and therapeutic target to improve outcomes for LUAD patients.
